# Degeneration of the Sensorimotor Tract in Degenerative Cervical Myelopathy and Compensatory Structural Changes in the Brain

**DOI:** 10.3389/fnagi.2022.784263

**Published:** 2022-04-04

**Authors:** Senlin Chen, Ying Wang, Xianyong Wu, Jianchao Chang, Weiming Jin, Wei Li, Peiwen Song, Yuanyuan Wu, Jiajia Zhu, Yinfeng Qian, Cailiang Shen, Yongqiang Yu, Fulong Dong

**Affiliations:** ^1^Department of Orthopedics, Department of Spine Surgery, The First Affiliated Hospital of AnHui Medical University, Hefei, China; ^2^Department of Radiology, The First Affiliated Hospital of AnHui Medical University, Hefei, China; ^3^Department of Medical Imaging, The First Affiliated Hospital of AnHui Medical University, Hefei, China

**Keywords:** DCM, DTI, corticospinal tract, gracilis and cuneatuc tract, VBM, TBSS, Wallerian degeneration

## Abstract

Degenerative cervical myelopathy is a progressive neurodegenerative disease, that has become increasingly prevalent in the aging population worldwide. The current study determined the factors affecting degeneration in the sensorimotor tract with degenerative cervical myelopathy and its relationship with brain structure. We divided patients into hyperintensity (HS) and non-hyperintensity (nHS) groups and measured the fractional anisotropy and apparent diffusion coefficients of the lateral corticospinal tract (CST), fasciculus gracilis and fasciculus cuneatus (FGC). Voxel-based morphometry (VBM) and tract-based spatial statistics (TBSS) techniques were used to estimate brain structure changes. Correlation of the modified Japanese Orthopaedic Association (mJOA) score, light touch, pinprick, motor score, and fractional anisotropy (FA) ratios of the CST at different levels were analyzed. Compared to healthy controls, the FA ratios of CST in the HS and nHS groups were decreased at all levels, and the apparent diffusion coefficient (ADC) ratio was increased only at C4/5 levels in the HS group. The FA ratio of FGC was decreased at the C3/4 and C4/5 levels in the HS group and only decreased at the C4/5 level in the nHS group. The ADC ratio was decreased only at the C4/5 level in the HS group. VBM analysis revealed that the volume of the precentral gyrus, postcentral gyrus, and paracentral lobule increased in patients compared to controls. TBSS analysis found no statistical significance between the sensory and motor tracts in white matter. The volume of clusters in HS and nHS groups negatively correlated with the C1/2 FA ratio of the CST. The results showed that the degeneration distance of the CST was longer than the FGC, and the degeneration distance was related to the degree of compression and spinal cord damage. Structural compensation and the neurotrophin family may lead to enlargement of the brain.

## Introduction

Degenerative cervical myelopathy (DCM) is a common form of non-traumatic spinal cord injury (Badhiwala et al., 2020) that is generally caused by compression of the cervical spinal cord *via* surrounding tissues, such as herniated discs, ossification of the posterior longitudinal ligament, calcification of the *ligamentum flavum*, abnormalities of the zygapophyseal and uncovertebral joints, and static and dynamic compression during cervical motion (Badhiwala et al., 2020; Yu et al., 2020; Tu et al., 2021). This progressive compression leads to apoptosis, inflammation, and vascular changes, which result in cell death, axonal degeneration, and myelin changes at the site of compression (Badhiwala et al., 2020; Seif et al., 2020). Demyelination is generally a chronic pathological process that is found in the injured segments and remotely from the site of injury (Freund et al., 2019), which leads to the anterograde or retrograde degeneration known as Wallerian degeneration (Levy et al., 2021), which is commonly found in the dorsal column and the lateral corticospinal tract of the spinal cord (Fischer et al., 2021). Although direct compression of the spinal cord may cause serious dysfunction, the Wallerian degeneration causing secondary degeneration, such as transsynaptic changes in the gray matter (David et al., 2019) of the axons, is a critical determinant of the final extent of neurological deficits. Therefore, the degree of demyelination on the cephalic and caudal sides of the injury is a potential factor to predict the final neurological outcome in patients with DCM.

Conventional MRI provides evidence of the degree of compression, injury, and intramedullary or extramedullary abnormal signals (Zhao et al., 2018; edema, hemorrhage, and injured segments). However, it is impossible to evaluate the microstructural changes during the process of neurodegeneration and compensation. Many quantitative magnetic resonance (qMRI) techniques have been used to study the spinal cord (Lommers et al., 2021; Savini et al., 2021), and diffusion tensor imaging (DTI) is one of the most commonly used research methods (McLachlin et al., 2021). DTI is a standard neuroscience technique that is more sensitive than conventional MRI in evaluating spinal cord injuries (Zhang et al., 2018), and it provides important information about structural integrity, including myelin formation, axon diameter, and fiber density (Kumar et al., 2021), which indirectly reflect the degree of degeneration and pathophysiological structural changes of the spinal cord *in vivo*. Previous studies found that conventional MRI did not show any abnormalities in the spinal cord, but DTI revealed reduced fractional anisotropy (FA) and increased apparent diffusion coefficient (ADC) values in these patients, which indicated potential injury to the spinal cord (Lee et al., 2015; Shabani et al., 2020; Singh et al., 2020). Therefore, DTI parameters were considered a more sensitive factor for the prediction of potential spinal cord injury in patients with DCM.

Secondary macrostructural or microstructural changes are not limited to the spinal cord but also include the brain, leading to the remodeling within the brain. Previous studies reported that the voxel-based morphometry (VBM) of cortical volume in spinal cord-injured patients showed significant gray matter volume (GMV) atrophy in sensorimotor system regions including the bilateral sensorimotor cortex (S1 and M1) and non-sensorimotor cortex, such as the supplementary motor area (SMA), paracentral gyrus, insular cortex, anterior cingulate cortex, and the middle frontal gyrus (Chen et al., 2017; Nardone et al., 2018; Wang et al., 2019), as well as multiple white matter (WM) tract damage in corticospinal and corticopontine tracts using tract-based spatial statistics (TBSS; Guo et al., 2019). However, few studies investigated whether patients with DCM suffered similar brain structural changes as patients with spinal cord injury (SCI). The present study determined that there might be compensatory changes in the brain after chronic injury in the cervical spinal cord, and this change may be due to brain remodeling. We detected the FA and ADC values at different levels of the CST and FGC above the spinal cord compression using DTI to evaluate the secondary microstructural changes on the cephalic side of the injury. TBSS and VBM were also used to investigate study the changes in white and gray matter, respectively.

## Materials and Methods

### Participants

**Figure 1:**
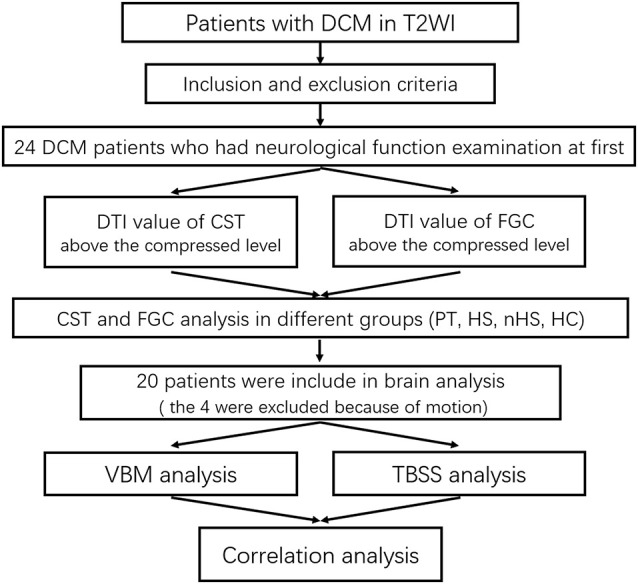
Flow diagram of the study.

Our study included 24 DCM patients (eight male and 16 female patients, with a mean age of 51.79 ± 6.79 years, and an age range from 40 to 64 years). The following inclusion criteria were used: (1) right-handedness; (2) MRI showed obvious cervical spinal cord compression with/without intramedullary hyperintensity; (3) no intracranial organic lesions or cognitive dysfunction; (4) no history of cervical and craniocerebral trauma or surgery; and (5) no contraindications for MRI examination. The following exclusion criteria were used: (1) alcoholism, smoking addiction, drug or coffee dependence, adiposity and malnutrition; (2) complicated with serious systemic diseases, such as hypertension, diabetes, heart disease and other basic or chronic disease histories; and (3) peripheral neuropathy (Figure 1).

It was previously reported that 58%–85% of DCM patients had hyperintensity on T2WI, which indicated edema, Wallerian degeneration, tissue loss, and necrosis, representing severe nervous system damage (Jannelli et al., 2020). Thus, the cervical DTI analysis divided patients into two subgroups: HS (four male and nine female patients, with a mean age of 52.23 ± 7.17 years, and an age range from 42 to 63 years) group and nHS (four male and seven female patients, with a mean age of 51.27 ± 6.35 years, and an age range from 40 to 64 years) group according to the presence or absence of intramedullary hyperintensity in T2WI. There was no significant difference in age or sex between the two subgroups (*P* > 0.05). Ten control subjects (four male and six female patients, with a mean age of 52.7 ± 6.67 years, and an age range from 42 to 62 years) were also recruited.

The brain structure analysis included 20 (eight male and 12 female patients, with a mean age of 52.4 ± 4.12 years, and an age range from 44 to 57 years) patients from the above 24 DCM patients, and four patients were excluded because of movement artifacts that could not be corrected by post-processing. The 20 patients were divided into two subgroups: HS (four male and six female patients, with a mean age of 52.1 ± 3.78 years, and an age range from 44 to 57 years) group and nHS (four male and six female patients, with a mean age of 52.7 ± 4.62 years, and an age range from 45 to 57 years) group according to intramedullary hyperintensity in the cervical spinal cord. There was no significant difference in age or sex between the HS, nHS, and healthy control groups (*P* > 0.05).

### Neurological Function Examination

Two experienced clinicians who were not involved in the study assessed neurological function using mJOA, ASIA^1^ (Kowalczyk et al., 2012; Table 1).

**Table 1:** Demographic and clinical information of DCM patients.

ID	Year/Y	Gender	Hyperintensity Y/N	mJOAscore	Light-touch/112	Pinprick/112	Motor/100
01	49	M	Y	16	110	109	100
02	53	F	Y	16	110	110	98
03	62	F	Y	15	110	110	96
04	46	F	Y	16	109	109	100
05	55	M	Y	13	106	108	92
06	42	F	Y	16	112	110	96
07	44	F	Y	15	110	108	99
08	52	F	Y	12	103	105	96
09	57	M	Y	16	108	109	100
10	62	M	Y	14	108	107	92
11	46	F	Y	15	108	108	98
12	48	F	Y	15	108	110	97
13	63	F	Y	15	108	108	97
14	52	F	N	18	112	112	98
15	55	F	N	15	110	110	99
16	54	F	N	14	108	109	96
17	57	M	N	16	109	109	98
18	64	F	N	18	112	110	100
19	47	F	N	16	112	110	100
20	46	F	N	16	110	110	100
21	40	M	N	16	110	110	100
22	50	M	N	17	110	110	100
23	54	M	N	16	110	111	100
24	45	F	N	15	108	111	97

### Image Acquisition and Processing

#### Cervical DTI Image Acquisition and Processing

**Figure 2:**
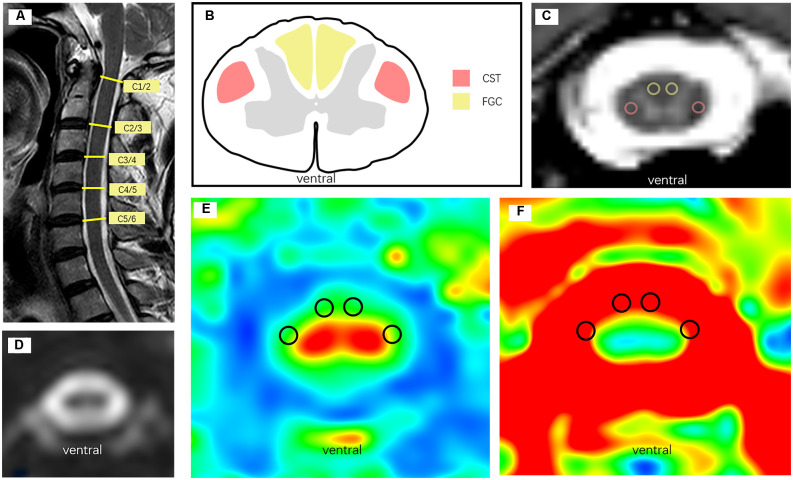
**(A)** Sagittal T2WI MRI of participants. **(B)** Schematic diagram of CST and FGC in the atlas of cervical anatomy, red represents CST and yellow represents FGC. **(C)** Manually draw ROI on axial T2WI image, red circle represents CST and the yellow represents FGC. **(D)** Structure image of DTI. **(E)** FA map of the axial cervical spinal cord. **(F)** ADC map of the axial cervical spinal cord.

A 1.5T magnetic resonance imaging (MRI) system (Philips Ingenia 1.5T, Holland) and dS head neck coil were used to cover the subjects’ head and neck. To reduce the disturbance of breathing and movement, all subjects were told to keep motionless and breathe calmly. The following settings were used: the single-shot spin echo echo-planar image (SS-SE-EPI) technique, the diffusion sensitivity gradient was taken in 15 different directions. *TR* = 3,000 ms, *TE* = 83 ms, slice thickness = 3 mm, field of view (FOV) = 300 mm × 300 mm, acquisition matrix = 100 × 98, gap = 0, and number of slices = 50, and total time = 376 s. The diffusion-weighted coefficient was *b* = 0 and 800 s/mm^2^, and 16 images were acquired after each scan (Figures 2A,C,D).

According to the principle of the blind, two radiology department doctors performed image processing using the MR Diffusion toolbox in a Philips Ingenia 1.5T MRI post-processing workstation. The diffusion tensor parameters of the cervical spinal cord were measured in all subjects at all levels of C1/2, C2/3, C3/4, and C4/5. The region of interest (ROI; 2 mm^2^) was manually placed on the combined DTI and T2WI images in the area of the corticospinal tract (CST) and fasciculus gracilis and fasciculus cuneatus (FGC) according to the anatomical atlas of bilateral symmetry (Wang et al., 2014; Alizadeh et al., 2018; Figures 2B,E,F). FA and ADC values were recorded in the FA and ADC maps, and the mean values on the left and right sides of each tract region were taken as the DTI parameters (FA and ADC values) at this level. To eliminate individual differences, DTI parameters at different levels were divided by the DTI values of the entire cervical C1/2 level, which were called DTI ratios, including the FA ratio and ADC ratio (Wang et al., 2017).

#### Brain 3D T1WI Image Acquisition and Processing

MR images were acquired using a whole-brain 3.0T MR system (General Electric 750 w, USA) with a 24-channel head coil. Earplugs were used to reduce scanner noise, and foam padding was used to minimize head motion. T1-weighted high-resolution three-dimensional structural images were obtained using a brain volume (BRAVO) sequence (echo time (TE) = 3.2 ms, repetition time (TR) = 8.5 ms, flip angle (FA) = 12°, field of view (FOV) = 256 mm × 256 mm, acquisition matrix = 256 × 256, slice thickness = 1 mm, no gap, number of slices = 188; Cai et al., 2021), and total time = 296 s. None of the participants exhibited abnormalities in brain structures.

Data processing was performed using Statistical Parametric Mapping (SPM) 8.0^2^ and executed in the MATLAB 2011a platform (Mathworks, Sherborn, MA). First, we processed the data *via* spm8 for bias correction and segmentation of 3D T1WI images. All images were segmented and classified into a number of tissue probability maps including GM, WM, and CSF. GM images obtained in the previous step were normalized to MNI space using a non-linear image registration tool (Diffeomorphic Anatomical Registration using Exponentiated Lie Algebra, DARTEL). The normalized GM component was modulated and smoothed using an 8-mm full width at half maximum (FWHM) Gaussian kernel which provided an ideal degree of increment in the signal-to-noise ratio (SNR) and conformation of MRI data to a normal distribution (de Moura et al., 2018). Second, covariance analysis (ANCOVA) with age and sex as nuisance variables was used to determine the significant difference in gray matter between patients (HS and nHS) and the control group (*P* < 0.05, false discovery rate correction for multiple comparisons). Two-tailed t-tests were used to assess the differences between groups at each voxel. Significant gray matter volume differences were superimposed on individual T1-weighted anatomical images for visualization. Finally, the cluster obtained from HS-HC and HS-nHS was saved as a “mask”. The volume used for correlation analysis was abstracted from all participants using the “Extract ROI Signals” in REST 1.8 (Resting State fMRI Data Analysis Toolkit^3^) with the masks described above. XjView^4^ and Brainnet Viewer^5^ (Xia et al., 2013) were used for displaying results.

#### Brain DTI Image Acquisition and Processing

Brain DTI data were acquired using a single-shot EPI sequence with *b* = (0.800) s/mm^2^; TR/TE = 10,000/74.2 ms, FOV = 256 mm × 256 mm, matrix = 128 × 128, FA = 90°, slice thickness = 3 mm, no interslice gap, 65 axial slices, total slices = 50 and total time = 700 s.

MRICRON^6^ software was used to convert the original DICOM files of all participants into the NIFTI format for processing by FSL software. The b0 images were extracted and aligned to the original data. To obtain the brain mask without a skull, the Bet tool in the FSL was used to divest the external brain tissue. Subsequently, we performed eddy current correction of all participants to correct tortuosity and movement in correction and used DTIFIT implemented in FSL to acquire fractional anisotropy (FA), mean diffusivity (MD), axial diffusivity (AD), and radial diffusivity (RD) for each participant. Finally, we calculated FA values of different brain areas based on the “rICBM_DTI_81_WMPM_FMRIB5” and “JHU_ICBM_tracts_maxprob_thr25_1 mm” standard templates.

### Statistical Analysis

All statistical analyses were performed using GraphPad Prism 9.0 (San Diego, CA, USA) software^7^. A two-sample t-test was used to analyze the age, CST, and FGC DTI ratios, and DTI values of tracts in white matter between the patient group (including subgroups) and healthy controls, and chi-square test was used for gender difference analysis. Pearson correlation analysis was used to analyze the correlation between the neurological function score and DTI values of the cervical spinal cord tract. D’Agostino-Pearson’s test was used to test normality for all data, the Mann-Whitney test was used for analysis when the data did not conform to a normal distribution, and the Spearman test was used for correlation analysis. *P < 0.05; **P < 0.01; ***P < 0.001; ns, no significance.

## Results

### FA and ADC Ratios of CST and FGC in PTs and HCs

**Figure 3:**
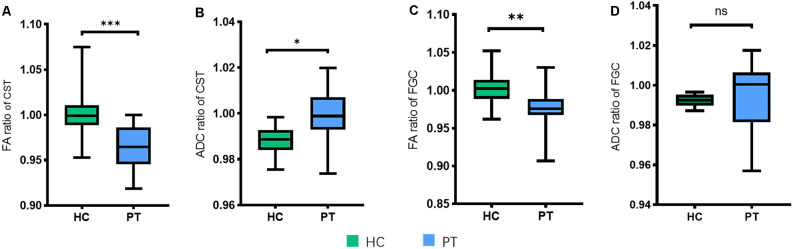
FA and ADC ratios in different groups. **(A)** FA ratio of the CST and the difference between HC and PT. **(B)** ADC ratio of the CST and the difference between HC and PT. **(C)** FA ratio of the FGC and the difference between HC and PT. **(D)** ADC ratio of the FGC and the difference between HC and PT (no significance, *P* = 0.8131). *P < 0.05;**P < 0.01; ***P < 0.001; ns, no significance.

To evaluate the degeneration of microstructure in patients with DCM, we assessed the FA and ADC ratios in the CST, which has a relationship with motor function, and in the FGC, which is associated with sensory function. The FA ratios of CST (*P* < 0.05) and FGC (*P* < 0.05) were reduced compared to the control group. The ADC ratio of the CST (*P* < 0.05) was increased in patients compared to controls, but no significant difference was found in FGC (*P* = 0.8131; Figure 3). These results revealed that the CST and FGC had axon degeneration in DCM patients.

### FA and ADC Ratios of the CST and FGC in Different Segments in PTs and HCs

**Figure 4:**
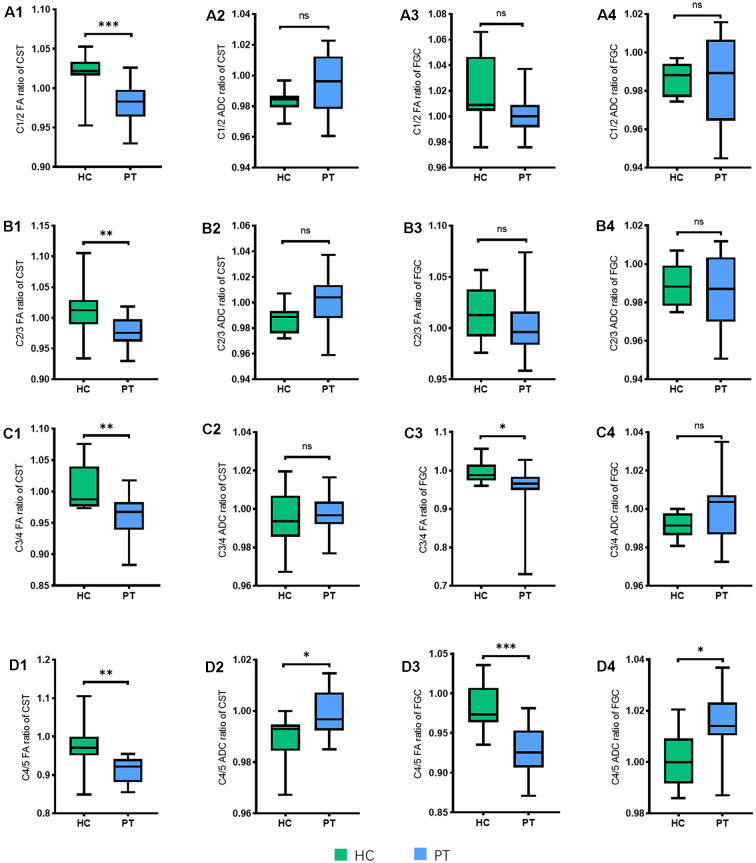
The FA and ADC ratios of the CST and FGC in different levels (from C1/2 to C4/5) between healthy controls (HC) and patients (PTs). **(A1–A4)** FA and ADC ratios of the CST and FGC in C1/2 (the P-value in ADC of the CST, *P* = 0.0636; in FA of the FGC, *P* = 0.0508; in ADC of the FGC, *P* = 0.8130). **(B1–B4)** FA and ADC ratios of the CST and FGC in C2/3 (the P-value in ADC of the CST, *P* = 0.0614; in FA of the FGC, *P* = 0.1724; in ADC of the FGC, *P* = 0.5227). **(C1–C4)** FA and ADC ratios of the CST and FGC in C3/4 (the P-value in ADC of the CST, *P* = 0.4704; in ADC of the FGC, *P* = 0.1361). **(D1–D4)** FA and ADC ratios of the CST and FGC in C4/5. *P < 0.05; **P < 0.01; ***P < 0.001; ns, no significance.

To observe whether the axonal degeneration occurred at different levels, we assessed the FA and ADC ratios in different segments from C1/2-C4/5, the results showed that the patients in DCM had a lower FA in the CST from C1/2 to C4/5 and a higher ADC in the C4/5 level (*P* < 0.05) compared to controls. The FA ratio of the FGC in the DCM group was only decreased at the levels of C3/4 and C4/5 (*P* < 0.05) compared to controls, and higher ADC ratios were observed in patients at the level of C4/5 (*P* < 0.05; Figure 4).

Based on a previous study, the degenerative distance from the compression site was closely related to the degree of Wallerian degeneration, which indicated that a more severe injury to the spinal cord may lead to a longer distance of degeneration. Because the compression of the spinal cord came from the front of itself, the anterior compression was more severe than the compression dorsally (Levy et al., 2021), which resulted in the different distances between the CST and FGC.

### FA and ADC Ratios of CST and FGC in Subgroups

**Figure 5:**
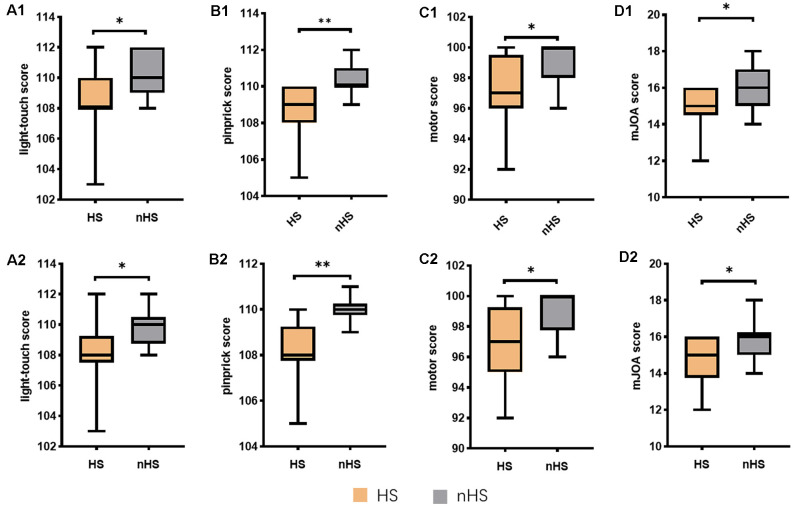
Neurological function scores between the HS and nHS. **(A1–D1)** Scores in cervical analyze. **(A2–D2)** Scores in brain analyze. Total light-touch score 112; total pinprick score 112; total motor score 100; total mJOA score 18. *P < 0.05;**P < 0.01.

Previous studies have reported that the presence of hyperintensity in the spinal cord on T2WI indicated worse injury to the spinal cord. To verify the assumption that the distance of degeneration was related to the degree of compression, we divided DCM patients into two subgroups, the hyperintensity (HS) and non-hyperintensity (nHS) groups based on the signal changes on T2WI. To confirm whether the HS groups had worse neurological function than the nHS groups, we evaluated the mJOA, light touch and pinprick scores in these groups. The results showed that the light touch, pinprick, and motor scores in the HS group were lower than the controls and nHS groups. Similar results were found in the nHS and control groups. The HS group had the lowest mJOA score compared to the nHS and control groups. In contrast, the nHS group had a higher mJOA score than the HS group but a lower mJOA score than the control group. The neurological function test suggested that HS patients had worse neurological function (Figure 5).

**Figure 6:**
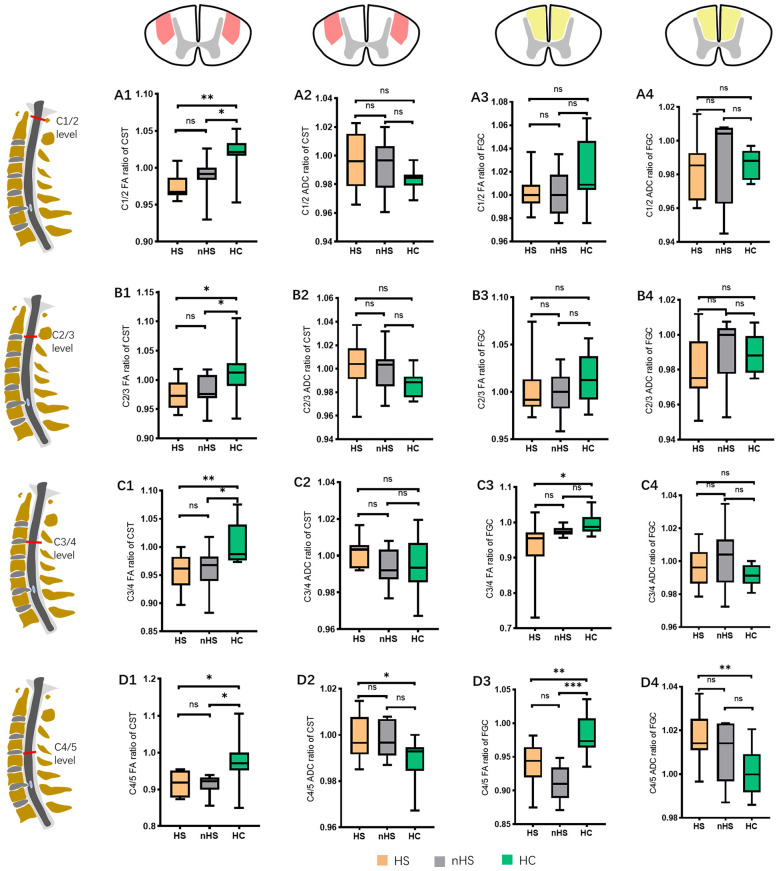
FA and ADC ratios of the CST and FGC from the C1/2 to C4/5 level. **(A1,A2)** The FA and ADC ratios of the CST in the C1/2 level between the HS, nHS and HC groups (no significance in the FA ratio between HS and nHS, *P* = 0.2388; no significance in the ADC ratio between HS and HC, *P* = 0.0546; nHS and HC, *P* = 0.1218; HS and nHS, *P* = 0.7666). **(A3,A4)** The FA and ADC ratios of the FGC in C1/2 level between the HS, nHS and HC groups (no significance in the FA ratio between HS and HC, *P* = 0.1127; nHS and HC, *P* = 0.1330; HS and nHS, *P* = 0.8244; no significance in ADC ratio between HS and HC, *P* = 0.4825; nHS and HC, *P* = 0.8454; HS and nHS, *P* = 0.4945). **(B1,B2)** The FA and ADC ratios of the CST in C2/3 level between the HS, nHS and HC groups (no significance in the FA ratio between HS and nHS, *P* = 0.7048; no significance in ADC ratio between HS and HC, *P* = 0.0760; nHS and HC, *P* = 0.0934; HS and nHS, *P* = 0.7559). **(B3,B4)** The FA and ADC ratios of the FGC in C2/3 level between the HS, nHS and HC groups (no significance in the FA ratio between HS and HC, *P* = 0.3029; nHS and HC, *P* = 0.1798; HS and nHS, *P* = 0.7663; no significance in the ADC ratio between HS and HC, *P* = 0.2556; nHS and HC, *P* = 0.9920; HS and nHS, *P* = 0.3235). **(C1,C2)** The FA and ADC ratios of the CST in C3/4 level between the HS, nHS and HC groups (no significance in the FA ratio between HS and nHS, *P* = 0.9449; no significance in the ADC ratio between HS and HC, *P* = 0.2094; nHS and HC, *P* = 0.8871; HS and nHS, *P* = 0.0632). **(C3,C4)** The FA and ADC ratios of the FGC in C3/4 level between the HS, nHS and HC groups (no significance in the FA ratio between nHS and HC, *P* = 0.0505; HS and nHS, *P* = 0.0799; no significance in the ADC ratio between HS and HC, *P* = 0.3254; nHS and HC, *P* = 0.0676; HS and nHS, *P* = 0.2442). **(D1,D2)** The FA and ADC ratios of the CST in C4/5 level between the HS, nHS and HC groups (no significance in the FA ratio between HS and nHS, *P* = 0.9139; no significance in the ADC ratio between nHS and HC, *P* = 0.0809; HS and nHS, *P* = 0.9262). **(D3,D4)** The FA and ADC ratios of the FGC in C4/5 level between the HS, nHS and HC groups (no significance in the FA ratio between HS and nHS, *P* = 0.0901; no significance in the ADC ratio between nHS and HC, *P* = 0.1860; HS and nHS, *P* = 0.3665). *P < 0.05; **P < 0.01; ***P < 0.001; ns, no significance.

We measured FA and ACD values in the HS and nHS groups from C1/2-C4/5. Compared to controls, HS and nHS patients had a lower FA ratio in the CST. Patients in the HS group had a higher ADC ratio of the CST in C4/5 than in controls. However, no statistically significant differences were found between the nHS and HC groups in the ADC ratios of the CST. In participants with HS, a lower FA ratio of FGC was found in C3/4 and C4/5, and a similar result was observed in C4/5 of the FGC FA ratio in nHS patients. Compared to controls, statistically significant differences in ADC ratios in the FGC were found in C4/5 of the HS group, but no statistically significant differences were noted in any segment in the nHS group (*P* = 0.1860). The results showed that CST had a longer distance of axon degeneration in the HS and nHS groups than in the controls. These results suggest that the degree of axonal damage is related to the distance of Wallerian degeneration (Figure 6).

### Brain Structural Changes in Different Subgroups

**Figure 7:**
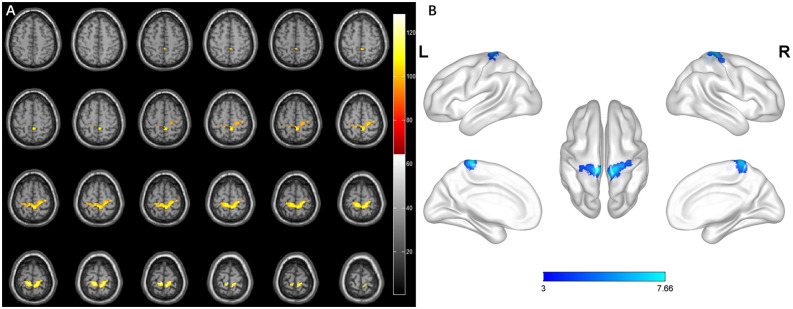
Whole-brain analysis results of regional differences between HS patients and HC. **(A)** Horizontal images made by xjView based on the result of VBM. **(B)** Three-dimensional images made by BrainNet Viewer based on the result of VBM.

**Figure 8:**
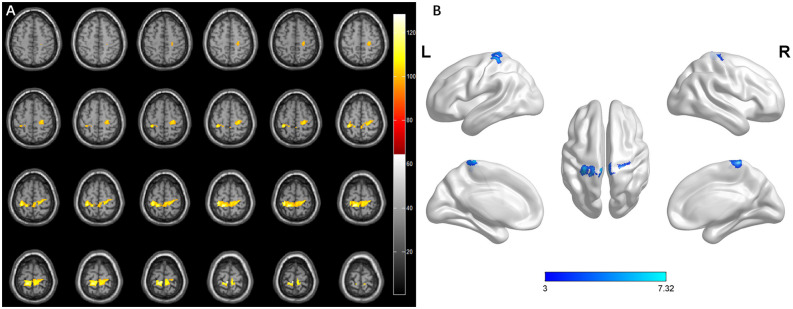
Whole-brain analysis results of regional differences between HS patients and nHS. **(A)** Horizontal images made by xjView based on the result of VBM. **(B)** Three-dimensional images made by BrainNet Viewer based on the result of VBM.

Previous studies showed that specific brain morphometric changes occurred after spinal cord damage (Goto et al., 2021; Wang et al., 2021). To estimate whether DCM patients had similar changes, we evaluated the brain structure in the HS and nHS groups using VBM. The results showed that the gray matter volume of HS patients exhibited an increase in the precentral gyrus which emits fibers to form pyramidal bundles that release and transmit voluntary motor impulses to lower motor neurons, compared to controls, and a similar change was noted in the postcentral gyrus, which receive the fibers from the thalamus and accurately feels pain, temperature, touch, pressure, position, and motor sensation from the contralateral half of the body. The cluster also contained the paracentral lobule and praecuneus. Patients with hyperintensity showed increments of GMV in the precentral gyrus, postcentral gyrus, paracentral lobule, and left praecuneus relative to patients with non-hyperintensity. However, no significant clusters were found between the nHS and HC groups (Table 2, Figures 7, 8).

**Table 2:** Regions showing significant difference of gray matter volume in different groups.

Group	Cluster size	Brain regions	Peak coordinates	*T*-value
	(voxels)	(No. of voxels)	(X, Y, Z)	
HS-HC	2,015	R-Precentral (431)	−6, −33, 73.5	7.66
		L-Precentral (16)		
		R-Postcentral (302)		
		L-Postcentral (151)		
		R-Paracentral Lobule (416)		
		L-Paracentral Lobule (443)		
		R-Precuneus (115)		
		L-Precuneus (77)		
HC-HS	No significant result
nHS-HC	No significant result
HC-nHS	No significant result
HS-nHS	2,461	R-Precentral (456)	−6, −33, 73.5	7.32
		L-Precentral (63)		
		R-Postcentral (227)		
		L-Postcentral (609)		
		R-Paracentral Lobule (485)		
		L-Paracentral Lobule (330)		
		L-Precuneus (111)		
nHS-HS	No significant result		

*Coordinates of the peak voxel in each cluster based on MNI Macro Anatomical label. GMV regions between groups at cluster-level FWE-corrected *p* < 0.05 (in HS-nHS, uncorrected *p* < 0.001, FWE Cluster = 2,461 voxels; in HS-HC, uncorrected *p* < 0.001, FWE Cluster = 2,015 voxels). Only regions showing significant differences between groups were included. For each cluster, the region showing the maximum *T*-value was listed first. Coordinates of the peak voxel (x, y, z) and volume (mm^3^) of clusters were also shown. No significant results were found in nHS-HS, HC-HS, nHS-HC, and HC-nHS*.

VBM analysis revealed that the brain structural changes in the gyrus of DCM patients with hyperintensity were associated with motor and sensory function, which may be caused by the degeneration of the CST and FGC in the cervical cortex. TBSS analysis found no statistical significance between the sensory and motor tracts in white matter between patients and the control group.

### Correlation Analysis Between Neural Function, DTI Values, and Brain Structure

**Figure 9:**
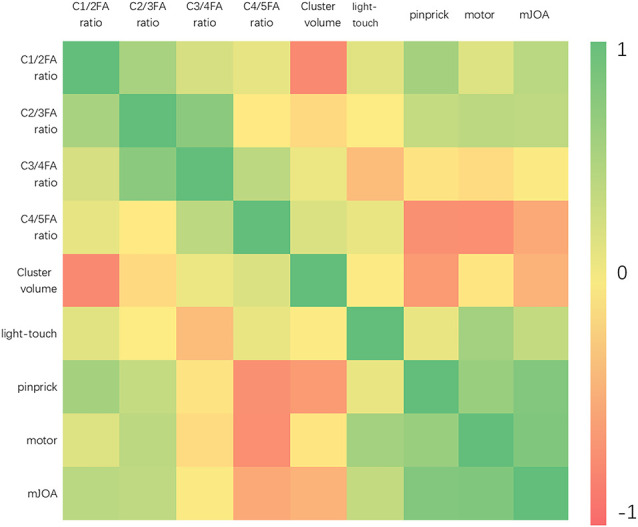
The heatmap demonstrates the relationship between the mJOA score, light touch, pinprick, motor score, and the FA ratios of the CST at different levels. The results showed the negative correlation between C1/2 FA ratio of the CST and cluster volume got from HS-HC in VBM analysis (*r* = −0.7421, *P* = 0.0350). The pinprick (*r* = 0.8058, *P* = 0.0157) and motor (*r* = 0.8198, *P* = 0.0127) scores were positively correlated with the mJOA score.

No statistically significant differences were found between any two factors of mJOA score, light-touch, pinprick, motor score, and FA ratios of the CST at C2/3, C3/4, and C4/5. The volume of clusters in the HS-HC negatively correlated with the C1/2 FA ratio of the CST. The pinprick and motor scores positively correlated with the mJOA score (Figure 9).

## Discussion

The current study focused on the degree and direction of sensory and motor tract degeneration with different degrees of compression injury in DCM patients by measuring the FA and ADC values of the corticospinal tract, gracilis, and cuneatus tract. The results showed that the degree of CST degeneration was more severe than the FGC, and the direction of degeneration may be retrograde to the brain. The volume changes of GM in patients with hyperintensity and non-hyperintensity were analyzed using VBM, and the results showed that the sensorimotor cortex increased in the HS compared to the nHS and HC.TBSS was used to analyze changes in white matter tracts, but no significant difference in sensory or motor tracts in white matter was found. These results indicated that long-term cervical spinal cord compression may be the cause of compensatory structural changes in the cerebral motor cortex in patients with DCM.

### Neurodegeneration and Compression

Anatomically, the anterior 2/3 of the spinal cord and the anterior part of the posterior column are infused by the anterior spinal artery, and the posterior 1/3 of the spinal cord is supplied by two posterior spinal arteries (Colman et al., 2015). When the blood supply is blocked during compression, chronic spinal cord injury may occur due to spinal cord ischemia. Because of arterial insufficiency, secondary spinal stenosis, or compression/stretch of intramedullary or extramedullary vessels during movement, the anterior horn of the spinal cord may be injured and result in multi segmental injury and loss of anterior horn cells (Luo et al., 2019). Post-mortem studies showed that spinal cord compression led to neuronal and axonal degeneration in the anterior horn (Yu et al., 2011). The combined effect of ischemia and compression led to Wallerian degeneration in the anterior horn and lateral funiculi (Akter and Kotter, 2018). As the disease progresses, the posterior horn of the spinal cord may also be affected, but it is smaller than that anterior horn (Akter et al., 2020; Levy et al., 2021). The microcircuit in the spinal cord of patients with DCM is damaged, which leads to disorders of nervous system regulation (Stachowski and Dougherty, 2021). All of these reasons will cause a more severe in the anterior than the posterior region.

Experimental evidence from spinal cord injury showed that Wallerian degeneration inducing axonal degeneration and demyelination of the spinal cord tracts were closest to the injury and decreased with increasing distance (Azzarito et al., 2020). The FA ratios of the CST and FGC were lower in patients with DCM than those in the control group. To further study whether there was a difference in the degree of degeneration in the CST and FGC, we measured the FA and ADC ratios of the CST and FGC at different levels in the patient group, we found that the distance of CST degeneration was greater than the FGC. These results showed that compression led to a degeneration of the CST and FGC, and the different compression degrees in the anterior and posterior spinal cord caused different degeneration distances which suggested the presence of a neurodegenerative gradient.

### Mechanism of Neurodegeneration in Hyperintensity and Non-hyperintensity

To assess the relationship between the degree of spinal cord damage and neurodegeneration distance, we evaluated the FA and ADC ratios of different segments of the CST and FGC in the HS and nHS groups. The results showed that the degeneration distance of tracts in patients with hyperintensity was longer than in patients without hyperintensity. This result indicated that the CST and FGC degenerated regardless of hyperintensity, and more serious damage to the tract was associated with a greater distance of degeneration.

A recent apoptosis theory believed that acute SCIwasreasonable for secondary degeneration and chronic demyelination in regions far from the lesions (Choi and Kang, 2020). Although DCM and SCI have different aetiologies, histological studies showed that spinal cord compression resulted in changes including interstitial edema, cytoplasmic reduction, and cavity formation (Akter et al., 2020) which is similar to SCI and induces demyelination and axonal degeneration. Chronic cord compression progression led to axonal destruction at the site of compression and cranial compression (Huber et al., 2018; Martin et al., 2018). Transsynaptic degeneration (TSD)may be related to the decreased excitability of α motoneurons in the spinal cord (Zheng et al., 2017), microangiopathy (Celik Buyuktepe et al., 2021), and pathological nerve remodeling (Lawlor et al., 2018). All of these factors may lead to apoptosis, inflammation, or ischemia, which ultimately mediate neuron damage and are responsible for remote degeneration in spinal cord tracts.

### Brain Structure Changes

As an important sensorimotor cortex, the volume of the precentral gyrus and postcentral gyrus transform after traumatic SCI (tSCI; Chen et al., 2019; Karunakaran et al., 2019; Wang et al., 2019). Some researchers consider that brain structure changes are caused by direct or secondary Wallerian degeneration, axon demyelination, or neuronal cell body atrophy after tSCI (Chen et al., 2017; Wang et al., 2019). However, there are relatively few studies on whether tract degeneration causes brain volume changed in DCM patients. The volumes of the precentral gyrus, postcentra lgyrus, and paracentral lobule were increased in patients compared to controls in our study. The TBSS analysis showed no significant difference in motor and sensory tracts in white matter between patients and controls. Therefore, we suggest that the retrograde influence of pathological changes in the injured spinal cord of DCM patients is finite and unlikely to directly result in brain structural changes.

### Mechanism of Increased Brain Volume From Macro and Micro Perspectives

Previous studies showed that the water content of brain tissue (Warntjes et al., 2018), diurnal fluctuation (Trefler et al., 2016), endocrine (Uher et al., 2021), or cardiovascular factors (Kappus et al., 2016) may influence brain volume. Different brain structures increase in response to changes in environments (Graeve et al., 2021). In contrast to the tSCI, spinal cord injury in DCM is a chronic process, and the main clinical symptoms of patients are abnormal strength and sensation in the extremities. To compensate for the partial loss of neurological function, the structural plasticity ability of neurons strengthened, such as increased length or diameter of the original synaptic branches, arousal of the original dormant synapses, or peripheral synaptic gemmation, which cause the sensorimotor area to expand and invade adjacent areas (Wang et al., 2019). The absolute volume or numbers of certain neurons may decrease in DCM, but additional space forintercellular connections may be obtained because of the larger intercellular distances, which expand the cell surface in the form of dendrite formation (Graeve et al., 2021). Astrocyte reorganization involves distal and proximal structures and cell bodies, which may generate a permissive environment for synaptic plasticity (Schmidt et al., 2021). Even the potential neuroprotective effects of preoperative conservative treatment may be possible, but this hypothesis must be investigated in further studies.

**Figure 10:**
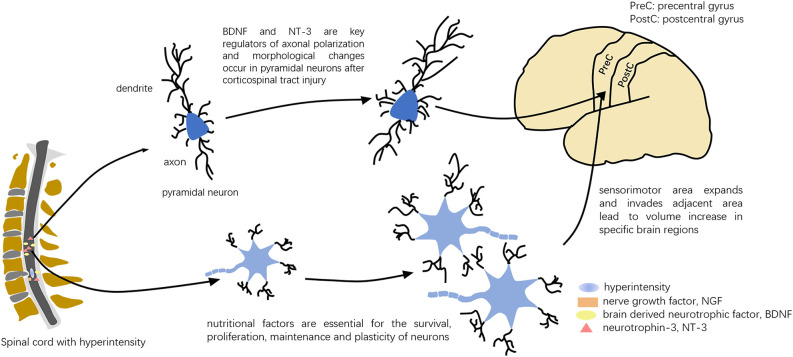
Possible pattern diagram for the influence of NFs. Various neurotrophic factors may mediate integrated growth and remodeling for cells and neurons.

The time profile of neural changes (acute onset vs. slowly developing symptoms) was the most obvious difference between the tSCI and DCM. At the same time, DCM patients could develop a central cord syndrome, which per definition was a tSCI. So we hypothesized that the neuropathological changes caused by the neurotrophin family may exist in DCM patients with hyperintensity which was similar to tSCI (Seif et al., 2020). The neurotrophin family (NF), such as nerve growth factor (NGF), brain-derived neurotrophic factor (BDNF) and neurotrophin-3 (NT-3), plays an important role in the recovery of the spinal cord after injury. BDNF and NT-3 were increased on the cephalic and caudal sides of the chronic compression of the spinal cord. These nutritional factors are essential for the survival, proliferation, maintenance, and plasticity of neurons (Di Carlo et al., 2019; Lima Giacobbo et al., 2019; Barker et al., 2020), and related to the early promotion of nerve regeneration, regulation of synaptic transmission and improvement of morphology (Xu et al., 2006; Volpicelli et al., 2014; Li et al., 2020). Morphological changes simultaneously occurred in pyramidal neurons in the primary motor cortex (M1) after corticospinal tract injury in animal experiments (Wrigley et al., 2009). Some studies suggested that BDNF and NT-3 were key regulators of axonal polarization in pyramidal neurons (Arikkath, 2020). Perhaps various neurotrophic factors converge to mediate the integrated growth and remodeling for certain cells and neurons, and consequently enlarge the volume (Figure 10).

The current study has several limitations, including a small sample size and the specific selection of patients, which may increase the risk of type I statistical errors and lead to additional factors that were treated as random variables. In our study, although we tried our best to avoid or correct the motion artifacts during scanning, this still could not completely rule out the false-positive results caused by this error and other unknown potential factors which required a larger sample size and more careful follow-up study. Second, the DTI measurement of cervical spinal cord tracts was performed manually, and it was hard to avoid manual measurement errors, so we adopted double measurements and consistency tests to improve their liability as much as possible. However, we have to mention that the partial volume effect of adjacent cerebrospinal fluid, abnormal tension patterns, and cerebrospinal fluid pressure in the spinal canal may also have an impact on DTI values (Banaszek et al., 2016). Therefore, some studies pointed out that the DTI parameters were great in terms of sensitivity (Kara et al., 2011; Banaszek et al., 2016; Rajasekaran et al., 2017), but less so in terms of specificity. Finally, the function of the brain in patients with DCM may be worth future study.

## Conclusion

Anterior compression of the spinal cord is more severe than posterior compression in patients with DCM, which results in the degeneration of the CST and FGC. The distance of degeneration is related to the degree of compression and spinal cord damage and the direction may be retrograde to the brain. Structural compensation and NFs may play important roles in the enlargement of the brain. DCM is a progressive neurological disorder that is caused by age-related spinal compression and degeneration. Our findings may contribute to the diagnosis and rehabilitation of chronic spinal cord injury caused by DCM, which may decrease the personal, family, and social economical burdens.

## Data Availability Statement

The raw data supporting the conclusions of this article will be made available by the authors, without undue reservation.

## Ethics Statement

The studies involving human participants were reviewed and approved by The First Affiliated Hospital of Anhui Medical University. The patients/participants provided their written informed consent to participate in this study.

## Author Contributions

SC, PS, and FD had full access to all of the data in the study and take responsibility for the integrity of the data and the accuracy of the data analysis. FD and PS: conceive and design the study. SC, YWa, and PS: drafting of the manuscript. SC, XW, WJ, WL, and JC: statistical analysis and graphic design. YY, YQ, JZ, and YWu: technical support. FD and CS: study supervision. All authors listed provided the design and made direct contribution to the work. All authors contributed to the article and approved the submitted version.

## Conflict of Interest

The authors declare that the research was conducted in the absence of any commercial or financial relationships that could be construed as a potential conflict of interest.

## Publisher’s Note

All claims expressed in this article are solely those of the authors and do not necessarily represent those of their affiliated organizations, or those of the publisher, the editors and the reviewers. Any product that may be evaluated in this article, or claim that may be made by its manufacturer, is not guaranteed or endorsed by the publisher.

## Footnotes

^1^ https://asia-spinalinjury.org/

^2^ http://www.fil.ion.ucl.ac.uk/spm/software/spm8

^3^ http://www.restfmri.net/forum

^4^ https://www.alivelearn.net/xjview/

^5^ https://www.nitrc.org/projects/bnv/

^6^ https://people.cas.sc.edu/rorden/mricron/dcm2nii.html

^7^ https://www.graphpad.com/scientific-software/prism/

## Abbreviations

DCM: Degenerative Cervical Myelopathy
DTI: Diffusion Tensor Imaging
HS: Hyperintensity
nHS: non-Hyperintensity
CST: Lateral Corticospinal Tract
FGC: Fasciculus Gracilis and Fasciculus Cuneatuc
VBM: Voxel-based Morphometry
MRI: Magnetic Resonance Imaging
FA: Fractional Anisotropy
ADC: Apparent Diffusion Coefficient
TBSS: Tract-Based Spatial Statistics
WD: Wallerian Degeneration
T1WI: T1 Weighted Image
T2WI: T2 Weighted Image
mJOA: modified Japanese Orthopaedic Association
ASIA: American Spinal Injury Association
TSD: Trans-synaptic Degeneration.
